# Superhydrophilic and Superhydrophobic Surfaces with Reversible Wettability Using Laser Processing

**DOI:** 10.1021/acsomega.5c11755

**Published:** 2026-05-07

**Authors:** Anustup Chakraborty, Mahantesh Khetri, Atchutananda Surampudi, Mool C. Gupta

**Affiliations:** Department of Electrical and Computer Engineering, 2358 University of Virginia, Charlottesville, Virginia 22904, United States

## Abstract

Laser surface microtexturing has become increasingly popular due to its low cost, scalability, automation, and lack of use of hazardous chemicals. Laser surface microtexturing can be used to control the wettability of various surfaces by varying surface roughness for a wide range of applications. In recent years, the reversible transition between superhydrophobicity and superhydrophilicity has gained significant attention due to a variety of applications, including self-cleaning, corrosion protection, anti-icing, microfluidics, water harvesting, and thermal management. The ability to manipulate liquid behavior facilitates processes like coalescence, rolling, and pinning. However, the current methods of making reversible superhydrophilic–superhydrophobic surfaces have several disadvantages, such as complex fabrication processes, use of high temperatures and chemicals, time-consuming, and high costs. This paper presents a facile, low-cost laser microtexturing method capable of inducing superhydrophilicity with a contact angle (CA) of less than 1°. Additionally, a thin poly­(dimethylsiloxane) (PDMS) coating can render the laser microtextured surface superhydrophobic with a CA of 169°. Furthermore, the thin PDMS layer can be easily removed by laser ablation, thereby restoring the superhydrophilic behavior, and allowing to achieve a reversible transition between superhydrophilicity and superhydrophobicity. As an example, the reversibility is shown for transparent glass surfaces and aluminum. Also, we demonstrate the laser method for the fabrication of selective areas having superhydrophilic and superhydrophobic properties selectively distributed over the surface.

## Graphical abstract

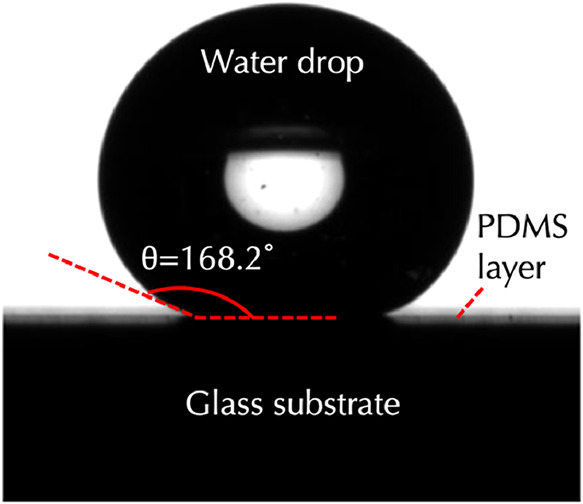

## 1 Introduction

When a water droplet is placed on a rough surface that exhibits superhydrophilic properties, a spontaneous spreading phenomenon with an almost negligible contact angle.^1,2^ This behavior can be explained by the classical Wenzel’s theory,^3–6^ which attributes the enhanced wettability to the presence of surface roughness, which is applicable to surfaces that are naturally hydrophilic. On the other hand, a superhydrophobic surface is characterized by a water droplet having a contact angle (C.A.) of greater than 150°, a contact angle hysteresis of less than 5°, and a roll-off angle of less than 5°.^7^ The superhydrophobicity can be explained by the Cassie–Baxter (C.B.) state.^8,9^ In the C.B. state, where air is trapped between the surface asperities, a droplet only partially wets the surface, resulting in a high contact angle with minimal hysteresis and surface adhesion.^8^

Laser-induced surface modifications have emerged as an effective approach for creating superhydrophilic surfaces, with their wetting characteristics significantly influenced by laser parameters such as pulse width, scanning speed, and frequency. In a study conducted by Lavieja et al.,^10^ a nanosecond laser was employed to induce superhydrophilicity on a polymer (acrylonitrile-butadiene-styrene) surface by adjusting the focal point. This process resulted in the formation of tiny ripples on the surface due to the intense localized heating generated by the laser. The reported contact angle was observed to be just below 1°. In a separate investigation, Pan et al.^8^ explored the impact of laser-generated surface microstructures on steel surfaces and their resulting superhydrophilic properties. They successfully introduced groove-like textures onto the surface by varying laser processing parameters. The measured contact angle exhibited fluctuations around 0°. Further endeavors are required to achieve greater consistency in contact angles while simultaneously enhancing the durability of these superhydrophilic surfaces.

Laser processing can also be used to create surface roughness to create superhydrophobic surfaces. Ta et al.^11^ investigated different direct laser processing conditions and parameters on the superhydrophobic properties of metals. Contact angles of greater than 150° were achieved. Another method was used by Mulroney et al.,^9^ where aluminum was microtextured by a nanosecond laser, followed by replication of the surface features on polycarbonate through hot embossing. Thereafter, PDMS was thermally cured on the microtextured polycarbonate sheet and pulled off, thereby yielding a superhydrophobic surface with CA above 150° and a roll-off angle (ROA) of below 2°. In another investigation by Chakraborty et al.,^12^ superhydrophobic surfaces were obtained by depositing laser-ablated polymer micro/nanoparticles over ceramic and metallic surfaces. Contact angles of greater than 150° were achieved by this method without using surface modification of the substrate. While these methods have showcased significant progress in achieving superhydrophobic surfaces with high contact angles, there exists untapped potential for further research into alternative techniques that could potentially yield even higher contact angles.

The transition of wettability can be achieved through various external stimuli that include ultraviolet (UV) irradiation, pH variations, electric fields, and heat treatment.^13,14^ In a study conducted by Zhou et al.,^15^ a highly responsive superhydrophobic TiO_2_ film was created through anodic oxidation. When exposed to UV irradiation, the film exhibited a rapid transition from superhydrophobicity to superhydrophilicity within 13 min. However, the reversal of this transition, from superhydrophilicity back to superhydrophobicity, required a much longer time of 100 h when heated at 60 °C in the absence of UV irradiation. Wang et al.^16^ introduced a technique for achieving a rapid, reversible wettability transition by creating a hierarchical micro/nanostructure on brass using alternating current (AC) etching. The initial superhydrophilic surface of the etched brass (EB) transformed into a superhydrophobic state after being modified with stearic acid for 1 min. Subsequently, when the modified EB surface was annealed at 350 °C for 5 min, it reverted to a superhydrophilic state. A significant drawback of this method is its reliance on elevated temperatures and the use of potentially hazardous chemicals. In another study done by Zhu et al.,^17^ a superhydrophobic coating composed of carbon nanotubes (CNTs) and polyethylene was created through a hot-pressing process, followed by silver (Ag) deposition and surface fluorination. The surface chemical composition and wettability of the coating were adjusted by employing air-plasma treatment and surface fluorination. These techniques enabled the control of surface properties, allowing for the reversible transition between superhydrophobicity and superhydrophilicity. One of the limitations of this process is its complexity, as it involves multiple steps and the use of various chemicals. Reversible wettability using plasma was also demonstrated by Wang et al.^18^ and Majhy et al.,^19^ but their methods are time-consuming, involve complex fabrication steps, and are limited by the choice of materials.

In recent years, researchers have made various efforts to achieve superhydrophilicity and reversible wettability using laser surface modification. Previous studies have reported improvement in wettability on laser-processed aluminum. Long et al.^20^ showed that picosecond-laser-textured aluminum gradually becomes superhydrophobic due to the adsorption of airborne organics. Yuan et al.,^21^ achieved rapid superhydrophobicity on femtosecond-laser-treated aluminum through brief heat treatment. In an investigation by Li et al.,^22^ a superhydrophobic micro/nanoscale hierarchical structure was fabricated on brass surfaces using laser ablation and subsequent heat treatment. The research demonstrated that a reversible change between superhydrophobicity and superhydrophilicity could be achieved within 4 h through a series of heating and reheating cycles. In a similar study by Yalishev et al.,^23^ it was found that laser-textured superhydrophilic metal samples can be transformed to display superhydrophobic behavior by storing them in a vacuum chamber for 6 h or in an atmosphere of organic compounds. Further, the transformation can be reversed by heating the samples at 300 °C for 30 min. Recent studies have further explored laser-assisted wettability modification and reversible wetting strategies across different substrates and coating approaches. For example, laser texturing combined with polymeric or chemical treatments has been shown to produce durable superhydrophobic and switchable surfaces.^24–27^ These works further establish the feasibility of laser-enabled topographic control and the utility of thin polymer overlayers for tuning apparent contact angles. It should be noted, however, that these methods are time-consuming and often require special storage conditions or high temperatures. Moreover, in these approaches, while it was stated that the surface became superhydrophilic following laser texturing, the exact measurement of the superhydrophilic contact angle was not adequately quantified, and it exhibited an increase over several days. In addition, these studies reported contact angles associated with superhydrophobicity that were below 165°.

In this work, we demonstrate picosecond-laser-induced micro/nanotexturing of Al 7075 alloy and glass substrates, followed by ultrathin PDMS overcoating, to realize extreme superhydrophilicity and laser-enabled switching to a superhydrophobic state. We selected aluminum (Al 7075) for this study because it is a widely used engineering alloy, is a convenient, machinable, and optically reflective metal at 355 nm wavelength that produces surface-confined heating with picosecond irradiation. These are conditions that favor formation of hierarchical micro/nano textures. Also, a picosecond laser was selected instead of a nanosecond laser primarily to achieve surface-confined energy deposition and to minimize thermal diffusion into the bulk material. In the picosecond regime, the laser pulse duration is shorter than or comparable to the electron–phonon relaxation time, which enables rapid energy localization near the surface and promotes controlled micro/nanoscale texture formation with reduced heat-affected zone, melting, and resolidification artifacts. In contrast, nanosecond pulses lead to stronger thermal diffusion, larger molten pools, and less controllable hierarchical structuring. Since the wettability transition in our work critically depends on preserving hierarchical micro/nano features, picosecond irradiation offers superior control and repeatability.

Through the creation of dense, pillar-like micro/nanostructures on the surface, we achieved superhydrophilicity with a water contact angle (CA) of less than 1°. This unique surface structure allows water to enter and spread, resulting in an exceptionally wettable surface. The distance wetted by the water drop at different time intervals on the surfaces of the textured samples was noted. This is an alternate way to measure superhydrophilicity. The superhydrophilicity remained stable without any noticeable changes in the contact angle over two months. Additionally, when a thin layer of poly­(dimethylsiloxane) (PDMS) was applied to these surfaces, they exhibited superhydrophobic behavior, characterized by a CA of approximately 169° and a roll-off angle (ROA) of less than 2°. Hierarchical surface micro/nanostructures covered by low surface energy PDMS impart the surface with superhydrophobicity properties. The PDMS coating can be easily removed using laser ablation to transform the surface back into its original superhydrophilic state. The presented method for achieving reversible wettable surfaces offers several advantages. It eliminates the requirement for high temperatures, and the use of hazardous chemicals, and it is faster and avoids more complex fabrication steps. The process can be completed within a short duration of just a few minutes and can be applied to a wide variety of materials. The surfaces appear to be stable over time. The contact angle reported in our study represents one of the highest reported values for superhydrophobicity (as shown in Table 1, in comparison with key papers from literature).

**Table 1:** Comparison of Contact Angles with Previous Works

refs	substrate	laser regime (pulse duration, wavelength)	surface treatment/coating	maximum reported static CA (°)	reversible wettability/cycling
^28^	PDMS	Femtosecond laser	Laser-modified PDMS (no additional coating)	∼165°	Not reported
^29^	Metals	Femtosecond laser	Laser-textured surface + ambient aging	160–165°	Limited stability tests
^30^	Transparent substrates	Ultrafast laser (fs–ps)	Laser texturing + hydrophobic treatment	∼150–160°	Not explicitly cycled
^13^	Glass/polymer	Pulsed laser ablation	Laser-ablated PDMS redeposition	>150°	Not reported
^31^	Multiple	fs/ps/ns	Various polymer and chemical coatings	Up to ∼165°	Mixed (study-dependent)
**This work**	Al 7075, glass	**Picosecond**, **355 nm**	**Laser texturing + ultrathin (few nanometer) PDMS coating**	**166–168°**	**Laser-enabled PDMS removal demonstrated**

Also, the method can be used to fabricate selective areas of superhydrophilic and superhydrophobic surfaces at the micron scale. We also demonstrate the reversible wettability transition for an optically transparent substrate like glass. However, the method is not fully reversible because it requires coating with PDMS every time.

### 1.1 Benchmarking with State of the Art

Superhydrophobic surfaces reported in the literature^28–31^ typically achieve static water contact angles above 150°, using approaches such as chemical etching plus coating, lithography, electrospinning, sol–gel processes, and laser texturing combined with low-surface-energy layers. Laser-based methods on metals generally report CAs in the ∼152–165° range depending on texture hierarchy and coating chemistry, comparable to the values obtained in this work. Compared with multistep wet chemical or template-assisted techniques, the present picosecond laser texturing combined with vapor-phase PDMS coating offers a mask-free, single-tool, and automation-compatible process with reduced procedural complexity and minimal chemical handling. Although laser systems involve higher initial capital cost, the overall process flow is simpler, scalable, and environmentally cleaner, making the approach competitive with state-of-the-art superhydrophobic fabrication methods in terms of performance, controllability, and practical implementation.

PDMS was selected in this study owing to its extensive applicability and proven performance as a low-surface-energy elastomer in microfluidics, soft lithography, biomedical coatings, and optical devices.^32–34^ PDMS study, which is a naturally hydrophobic material, has been reported previously. Its chemical stability, optical transparency, and ease of uniform deposition make it ideal for conformal coating on laser-textured micro/nanostructures.^35,36^ With a surface free energy of about 20–24 mN/m, PDMS imparts strong water repellency comparable to other fluoropolymers but is easier to process, less toxic, and adheres well to metals and glass.^37^ Although mechanically softer and less wear-resistant than other fluorinated materials, PDMS can be easily removed by mild laser irradiation, enabling a simple, reversible transition between superhydrophilic and superhydrophobic states. Furthermore, the chemical durability of PDMS coatings has been extensively reported in previous studies. PDMS exhibits excellent resistance to mild acids and bases due to its Si–O–Si backbone, which remains stable under a wide pH range (2–11) and moderate temperatures. Mulroney and Gupta^9^ demonstrated that PDMS-based superhydrophobic films retained their high contact angles after exposure to aqueous acidic and basic environments for several hours. Similarly, Ma et al.^38^ and Wang et al.^39^ observed negligible degradation in the wettability and morphology of PDMS-modified surfaces after prolonged immersion in corrosive media. These results indicate that PDMS provides reliable chemical and corrosion resistance without requiring additional fluorination or protective layers, making it a suitable and robust choice for reversible wettability applications.

## 2 Experimental Section

### 2.1 Materials

The substrates used were microscope glass slides (Length = 3 in.; Width = 1 in.; Thickness = 0.04 in.) by Amscope, recrystallized silicon-carbide ceramic sheets (Length = 3 in.; Width = 3 in.; Thickness = 0.125 in.), and aluminum alloy (Al 7075) samples (Length = 1 in.; Width = 1 in.; Thickness = 0.07 in.) polished down to roughness of less than 1 μm. The chemical composition by weight of Al 7075 was Zn = 5.4%, Cu = 1.42%, Mn = 0.12%, Mg = 2.42%, Fe = 0.42%, Cr = 0.21%, Ti = 0.11%, Si = 0.13%, and Al = 89.77%. The aluminum was purchased from McMaster-Carr. Poly­(dimethylsiloxane) (PDMS) SYLGARD 184 Silicon Elastomer was purchased from Dow Corning and used to coat the laser microtextured glass and aluminum samples.

### 2.2 Sample Preparation Procedure

To microtexture the glass and aluminum, a 355 nm wavelength picosecond pulsed laser from Spectra-Physics IceFyre 355–30, operating at 1 MHz, at 30 W average power and pulse energy of 20 μJ was used. The pulse width of the picosecond laser was 10 ps. A focused laser beam of full width at half-maximum (FWHM) size of 50 μm was used. The laser beam was scanned using the Sino-Galvo SG7210 system. High Dynamics PIMag linear *XY* stage was used for mounting the samples. The experimental setup of the laser is shown in Figure 1. The UV laser was chosen for this experiment because metals and ceramics are highly reflective at most of the laser energy from longer wavelength lasers. Additionally, shorter pulse-width lasers are good candidates for laser microtexturing.^40,41^ The laser fluence used for the experiments was 4.25 J cm^–2^. The laser beam scan speed was maintained at 200 mm/s. For each laser fluence, the side-to-side overlap between the lines was 50%.

**Figure 1:**
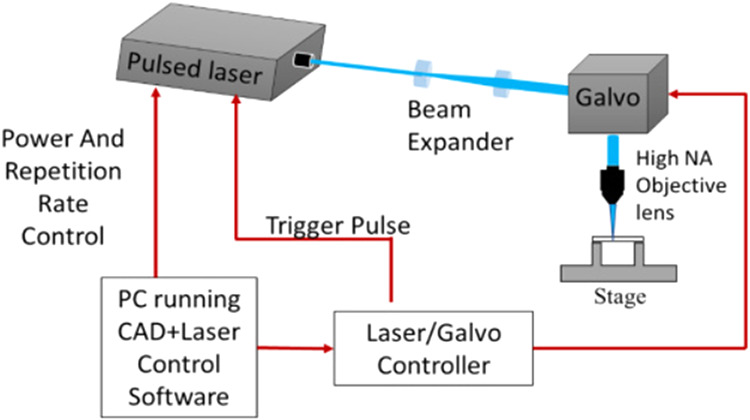
Experimental setup for UV picosecond laser processing.

Following the laser microtexturing process, a thin layer of PDMS was applied to the samples. The PDMS utilized in this experiment consisted of a two-part solution mixture. Ten parts of the polymer were thoroughly mixed with one part of the curing agent to prepare the PDMS solution. Once prepared, the solution was poured into a circular glass Petri dish measuring 90 mm × 15 mm. The dish was then placed on a hot plate heated to 115 °C for 15 min. This produced vapor-phase transport and subsequent condensation of volatile low–molecular-weight siloxane oligomers onto the textured substrates.^42^ The textured samples were positioned with the textured side facing downward and resting on the edges of the Petri dish.

Figure 2 depicts a schematic representation of the experimental setup. Following the PDMS deposition, the samples underwent laser treatment to remove the PDMS coating, leading to a reversible transition between superhydrophobic and superhydrophilic surface properties.

**Figure 2:**
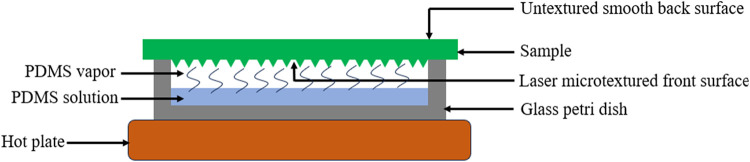
Schematic of the PDMS deposition setup.

### 2.3 Water Flow Testing Procedure

After the laser treatment, each of the samples was inclined at an angle of 45°, and a drop of water (10 μL) was dropped on one end of the textured area. Then, the movement of the water was observed as the water flowed across the length of the textured area. As a reference, a drop of water (10 μL) was also dropped on a smooth, untextured area of each of the samples, and the water flow was observed. The time taken by the water to traverse/completely wet the length of the textured area was observed with a camera and recorded.

### 2.4 Surface Characterization

The surface morphology of the laser microtextured samples was characterized using FEI Quanta 650 Field Emission SEM 3D optical profile measurements were done using an Olympus LEXT OLS4000 3D Laser Microscope to understand the variation in the microtexture heights, peak-to-valley spacing, and the density and uniformity of the features. To analyze the chemical composition and its percentage, Energy-Dispersive X-ray Spectroscopy (EDS) was performed on the samples both before and after laser microtexturing and subsequent PDMS deposition. The EDS analysis was conducted using FEI Quanta 650 Field Emission Scanning Electron Microscope (SEM). The water contact angle measurements were done using the Ramé–Hart Model 250 Goniometer. 10 μL water droplets were used for all contact angle measurements.

Optical images of the superhydrophobic-superhydrophilic patterned surface were taken using Hirox RH-8800 Light Microscope.

### 2.5 Optical Characterization Procedure

The optical characterization of the laser microtextured glass surface covered with PDMS was performed by transmitting light across the solar spectrum range (350–2500 nm) and detecting the transmitted light using a power measurement meter (Thorlabs S302C thermal power sensor).

### 2.6 Raman and FTIR Measurements

Raman spectra were acquired using a 405 nm excitation source with a backscattering geometry (instrument: Renishaw InVio; objective: 50×, grating: 1800 l/mm; integration time: 30 s; accumulation: 1). Measurements were performed on two sets of samples: (Set 1) bare glass, laser-textured glass, and PDMS deposited on textured glass; (Set 2) bare aluminum, laser-textured aluminum, and PDMS deposited on textured aluminum. FTIR measurements were performed using the Thermo Fischer Nicolet iS50 spectrometer with following specifications: specular reflectance accessory, diamond tip, 3000–600 cm^–1^ mid-IR range, 4 cm^–1^ spectral resolution, averaging of 64 scans per spectrum. A polished aluminum substrate was used as the reflectance reference (100% baseline). The PDMS-coated aluminum sample was then measured under identical optical geometry. The obtained reflectance spectra were expressed as %*R* and further processed using OMNIC software (Thermo Fischer Scientific) to apply Kramers–Kronig and baseline corrections.

### 2.7 Numerical Simulations/Modeling

To understand transient laser–material interactions during picosecond surface microtexturing, a Hyperbolic Two-Temperature (HH2T) model was solved in one dimension using an explicit finite-difference scheme.^43^ The model treats electrons and the lattice as coupled subsystems that exchange energy through electron–phonon coupling. Temperature-dependent electron heat capacity, electron–phonon coupling coefficients, and thermal conductivities were taken from reported literature for aluminum.^44^ The incident laser temporal profile was modeled as a single Gaussian pulse with 10 ps FWHM, centered at 30 ps, and a wavelength of 355 nm. Incident fluences between 0.5 and 2 J cm^–2^ were simulated to match experimental conditions.

## 3 Results

### 3.1 Laser Microtexturing Process and Superhydrophilicity

Figures 3 and 4 show the variation of the surface morphology with a changing number of laser scans. It can be seen that at a higher number of scans, the ablation is much more significant. Increasing the number of scans also results in a more well-defined surface microtexture.^41^ Moreover, scanning in different directions results in a more pronounced laser effect, resulting in increased depth and decreased spacing between the features.

**Figure 3:**
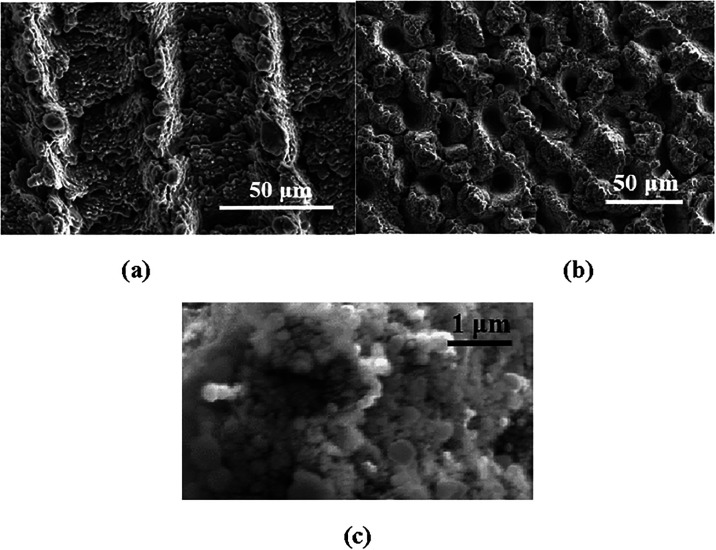
Laser micro/nanotextured aluminum sample at a fluence of 4.25 J cm^–2^. (a) One scan (0°), (b) three scans (0°, 90°, and 45°), (c) magnified image of the “three scans” surface showing nanostructures.

**Figure 4:**
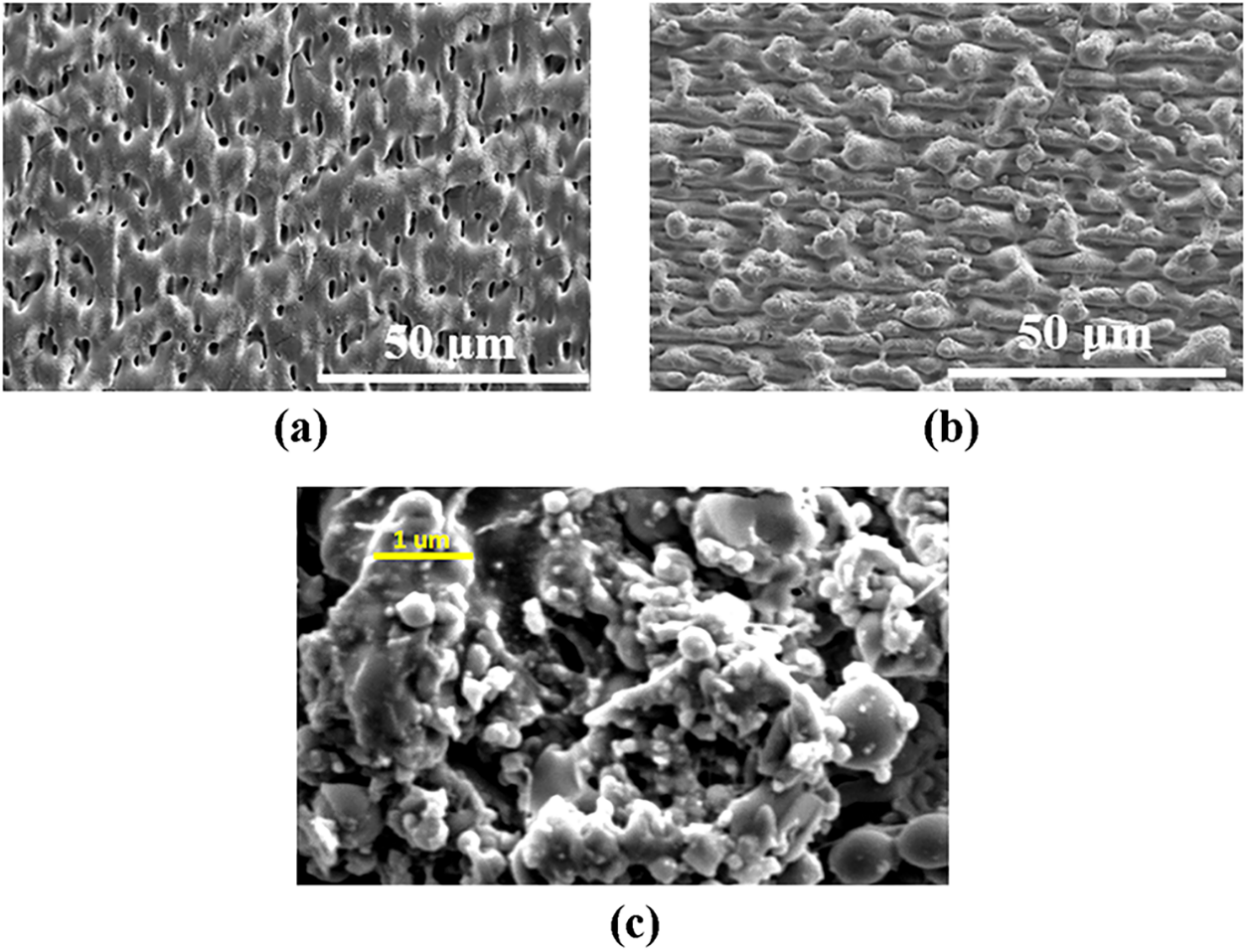
Laser micro/nanotextured glass sample at a fluence of 4.25 J cm^–2^. (a) One scan (0°), (b) three scans (0°, 90°, and 45°), (c) magnified image of the three scans surface showing nanostructures.

Figure 5 shows the superhydrophilic properties induced by laser surface microtexturing. The water contact angle (CA) on glass and aluminum surfaces microtextured with one scan was found to be 17.2° ± 1.8° and 21.2° ± 1°, respectively. On the other hand, glass and aluminum surfaces textured with three scans had a CA of less than 1°. We note that in the subdegree regime, the practical resolution of optical sessile-drop contact-angle measurements is typically on the order of ∼0.3–1°, limited by image resolution, droplet spreading, and evaporation. The values reported as <1° indicate extreme superhydrophilicity rather than exact absolute angles. The contact angles of laser microtextured glass and aluminum are given in the caption of Figure 5. The greater wetting behavior observed at a higher number of scans can be attributed to more pronounced surface features and greater depth of the grooves, resulting in increased penetration and spreadability of the water droplet. The superhydrophilicity remained stable without any noticeable changes in the contact angle over two months.

**Figure 5:**
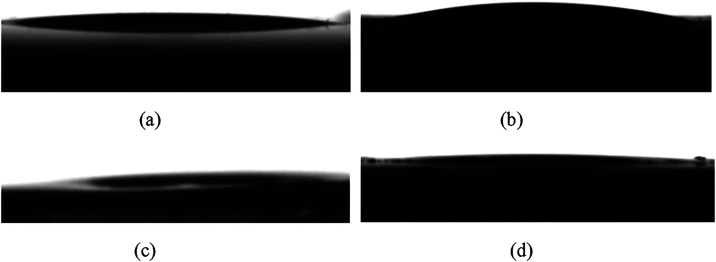
Water drop sitting on the one scan surface of (a) glass (CA = 17.2° ± 1.8°) and (b) aluminum (CA = 21.2° ± 1°). Water drop sitting on the three scans surface of (c) glass (CA < 1°) and (d) aluminum (CA < 1°).

Modifying the surface texture has a direct impact on the spreading behavior of water, influencing the speed of water flow. It has been observed that increasing the average feature height leads to a higher rate of water flow compared to surfaces with a lower average feature height. The length and width of the textured surface that was considered for the water flow experiment are 3 and 1 cm, respectively, and the measurements of the time taken to traverse/wet were done in steps of 0.5 cm, 1 cm, 1.5 cm, 2 cm, 2.5 cm, and 3 cm along the length for the surface with one and three scans. The same measurements were taken for the untextured surfaces as well. Thereafter, the velocities were calculated at each interval length. Compared to an untextured surface, there is a significant increase in the velocity for both glass and aluminum. The velocity of the water at different intervals of length for both glass and aluminum has been plotted in Figure 6. Figure 7 shows the position of the water drop with time on a textured (three scans) aluminum surface.

**Figure 6:**
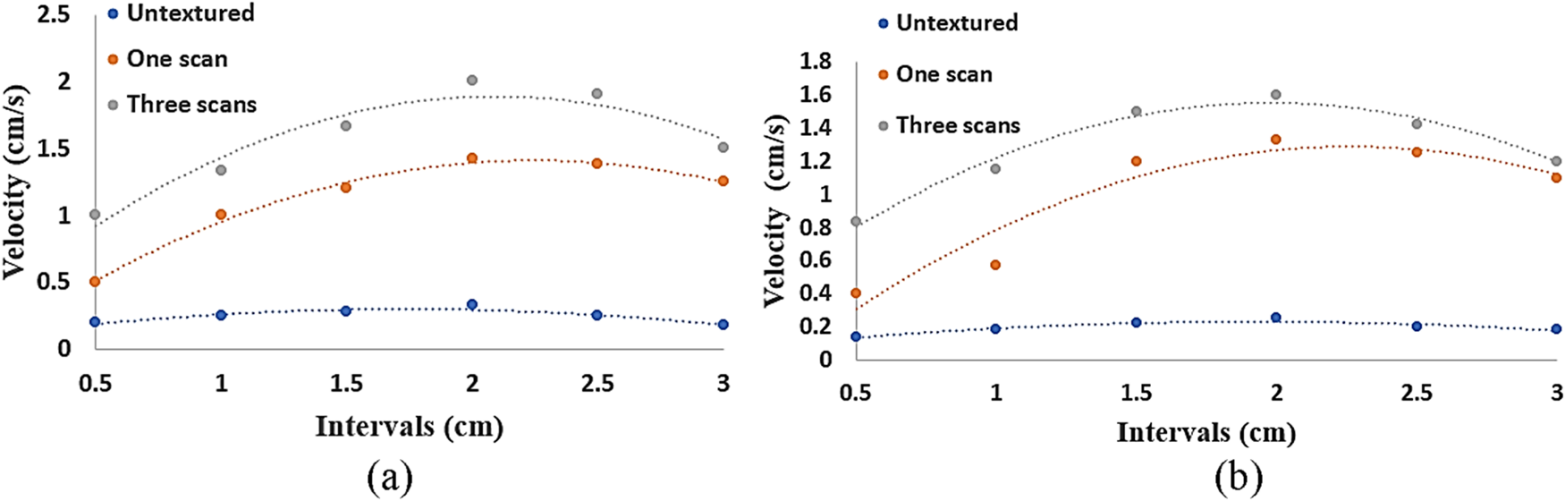
Velocity of water at different distances on the laser microtextured surface of (a) aluminum and (b) glass.

**Figure 7:**
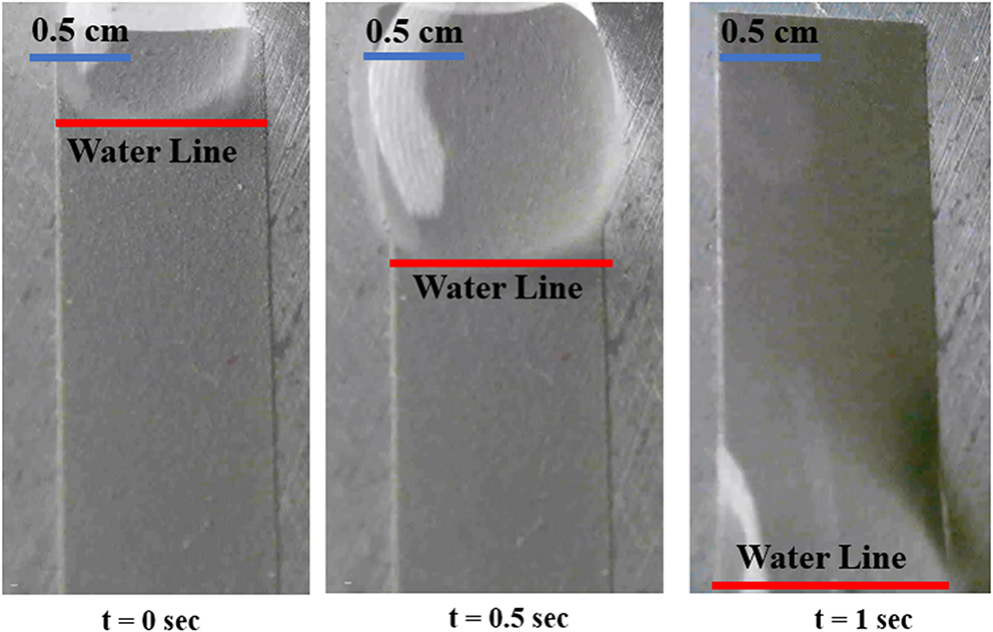
Flow of a water drop on a textured (three scans) aluminum surface.

Figure 8 displays the optical profile of the laser microtextured aluminum surface obtained through three scans. It can be observed that the maximum peak height reaches approximately 70 μm, while the average feature height measures around 55 μm.

**Figure 8:**
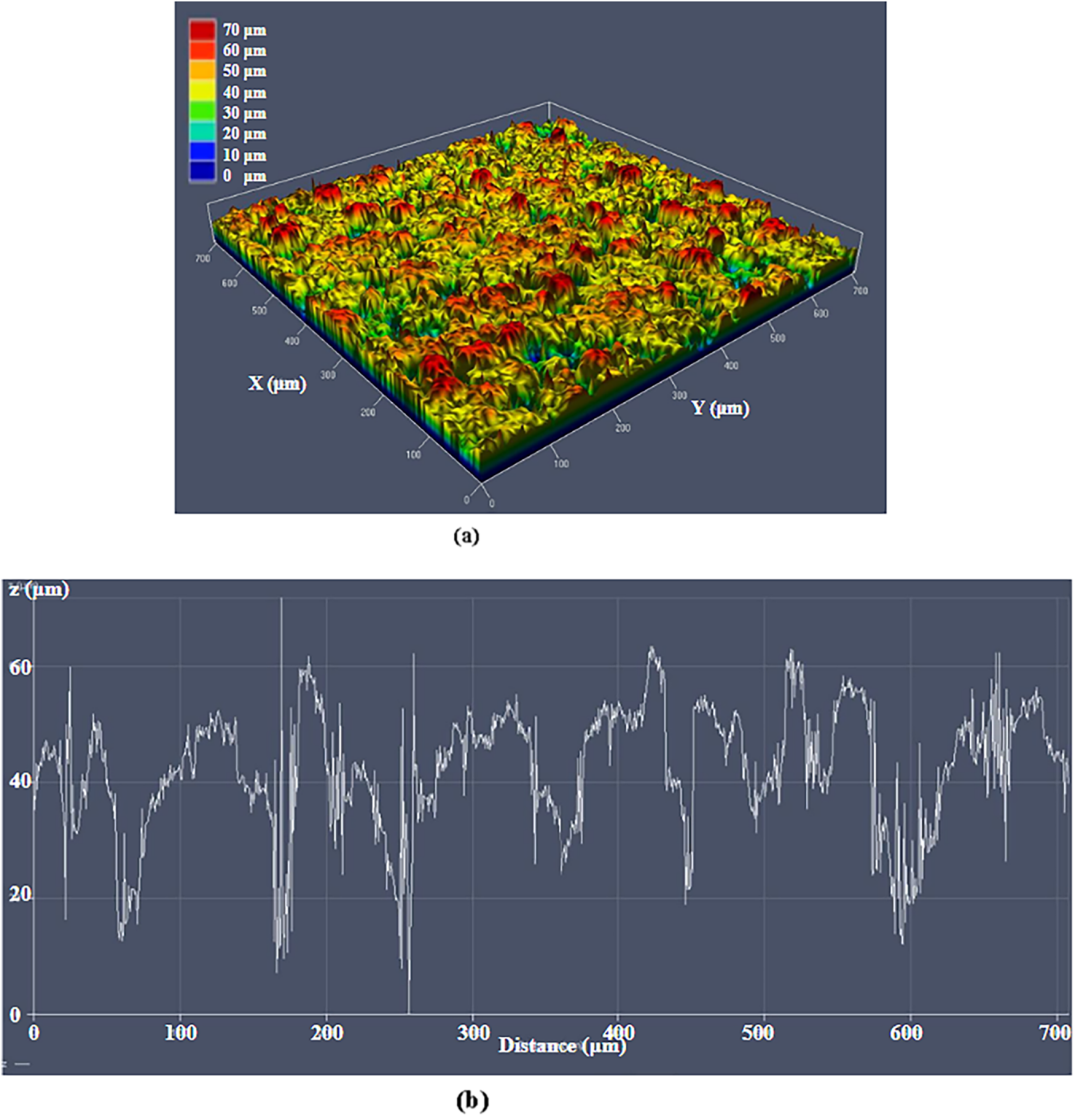
(a) 3D surface profile of the laser microtextured aluminum three scans surface, (b) 1D surface graph showing the surface variations of the laser microtextured aluminum three scans surface.

Figure 9 displays the optical profile of the laser microtextured glass surface obtained through three scans. It can be observed that the maximum peak height reaches approximately 60 μm, while the average feature height measures around 50 μm.

**Figure 9:**
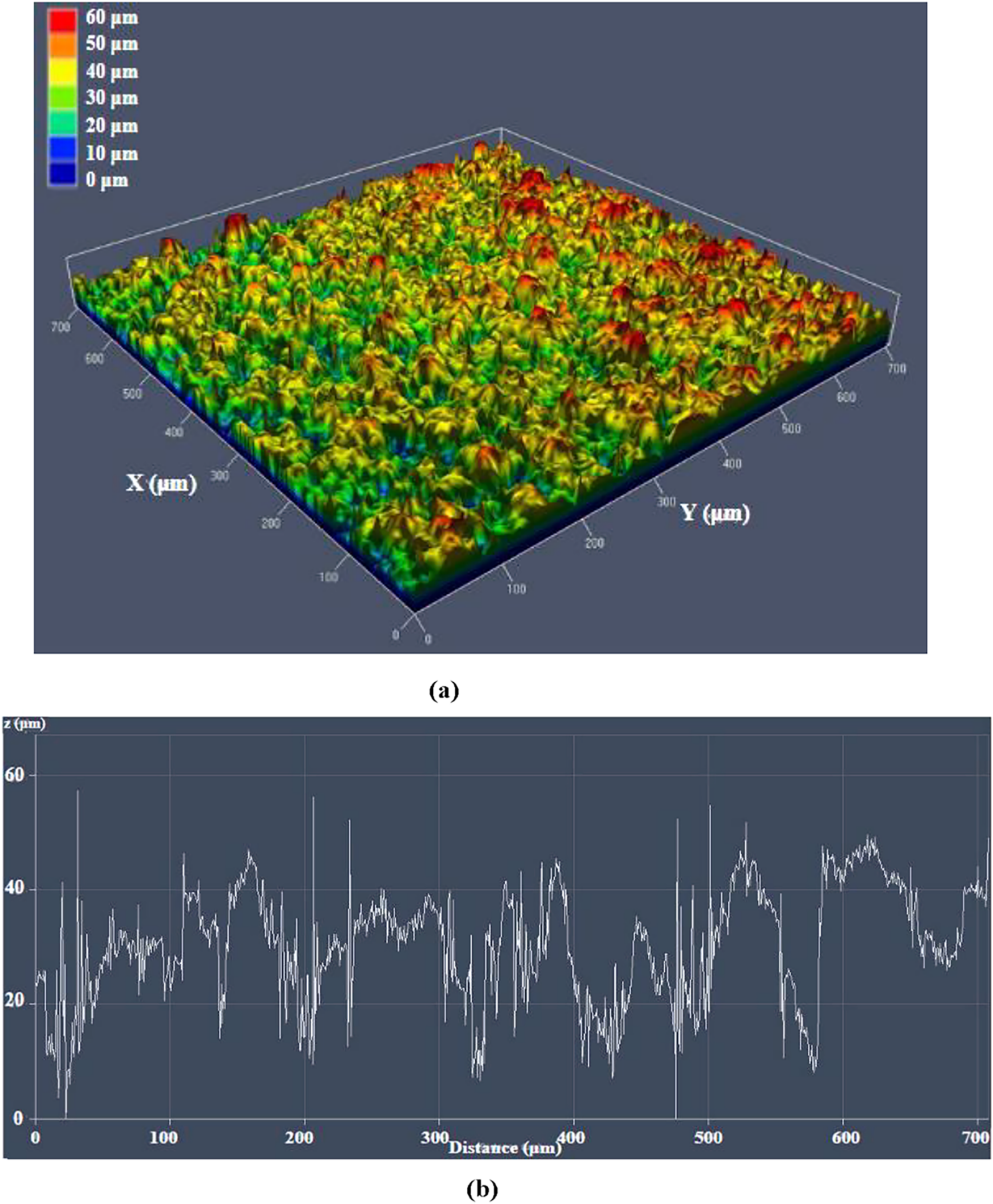
(a) 3D surface profile of the laser microtextured glass three scans surface, (b) 1D surface graph showing the surface variations of the laser microtextured glass three scans surface.

#### 3.1.1 Thermal Simulations of Laser-Material Interaction

Figure 10(a) illustrates the variation of surface lattice temperature with time for absorbed fluences ranging from 0.5 to 2 J cm^–2^. The temperature increases rapidly within ≈30–40 ps due to energy transfer from the excited electrons to the lattice. The peak surface temperature exhibits an almost linear dependence on absorbed fluence, rising from ≈2 × 10^3^ K at 0.5 J cm^–2^ to ≈9 × 10^3^ K at 2 J cm^–2^. At absorbed fluences ≥1.5 J cm^–2^, the temperature exceeds thermodynamical ablation threshold, 0.9 *T*_c_ (≈6030 K), suggesting the onset of melting and surface ablation.^45,46^ The corresponding depth-dependent temperature distribution shown in Figure 10(b) indicates that the heating is strongly confined to the top <100 nm of the surface. The temperature decreases sharply beyond this region due to aluminum’s high reflectivity, limited optical absorption depth, and high thermal conductivity. It should be noted that the thermal simulations were performed up to an incident fluence of 2 J cm^–2^, which already corresponds to surface lattice temperatures exceeding the ablation threshold for aluminum (0.9 *T*_a_ ≈ 6030 K). The experimentally employed fluence of 4.25 J cm^–2^ thus operates well within the ablation regime. Since the two-temperature model is most reliable up to the onset of material vaporization, the simulation range was deliberately limited to 2 J cm^–2^ to maintain physical accuracy. Although this range corresponds to an ablation depth below ≈80 nm per pulse, it effectively captures the dominant thermal dynamics governing ablation initiation and aligns with the experimentally observed cumulative ablation depths discussed in this work. Under the scanning parameters used (1 MHz repetition rate, 200 mm s^–1^ scan speed, and 50 μm beam diameter), the pulse-to-pulse spacing is approximately 0.2 μm, corresponding to ≈99.6% overlap between successive pulses. This results in ≈250 pulses per point per line and ≈500 pulses per point when accounting for the 50%-line overlap. After three passes, each surface location experiences ≈1500 overlapping pulses with spatially varying absorbed fluences due to the Gaussian intensity distribution. Consequently, the experimentally observed 50–70 μm-deep hierarchical microstructures (Figure 8) remain consistent with the predicted cumulative ablation depth expected from successive ultrafast pulse interactions at 1 MHz, confirming efficient energy accumulation and controlled surface structuring.

**Figure 10:**
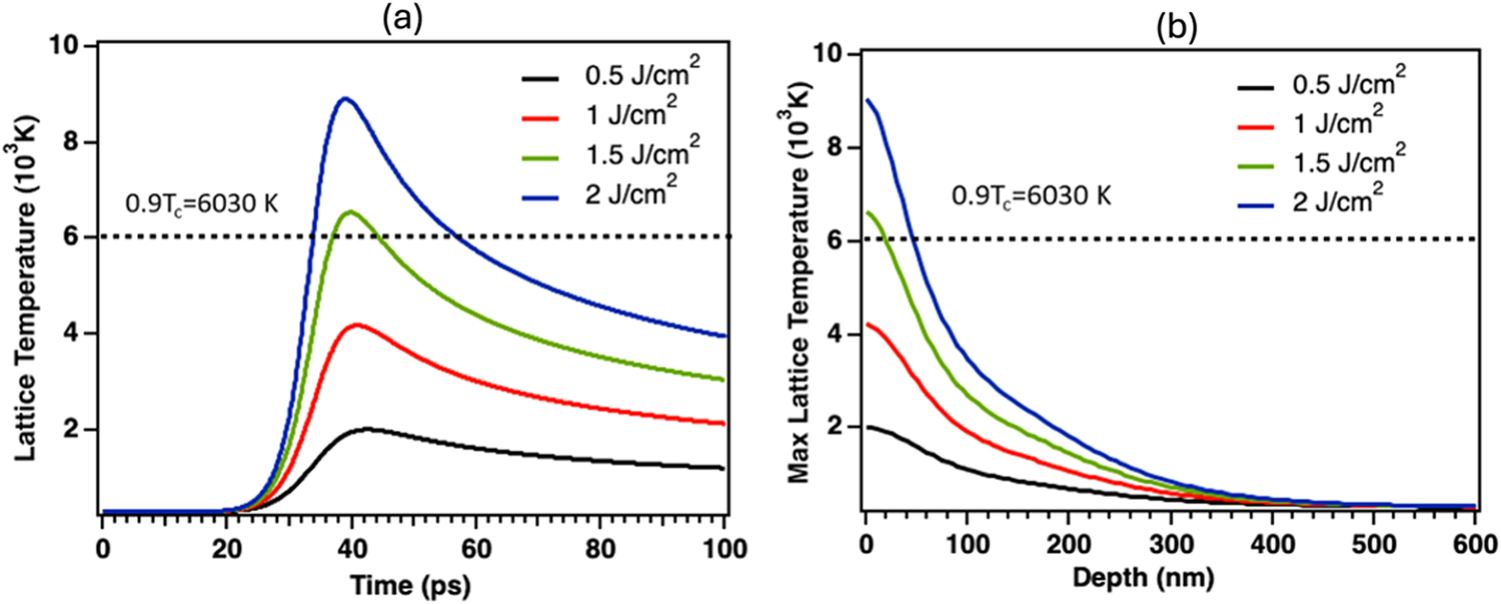
(a) Simulated surface lattice temperature as a function of time, (b) maximum lattice temperature as a function of depth for aluminum for incident fluences of 0.5, 1, 1.5, and 2 J cm^–2^ (Gaussian 10 ps FWHM beam centered at 30 ps).

### 3.2 PDMS Deposition and Superhydrophobicity

Figure 11 shows the superhydrophobic properties displayed by the one-scan laser microtextured glass and aluminum surfaces after PDMS was deposited on them. Figure 12 shows the superhydrophobic properties displayed by the three scans of laser microtextured glass and aluminum surfaces after PDMS was deposited on them. The contact angle results are shown in the caption of Figure 12.

**Figure 11:**
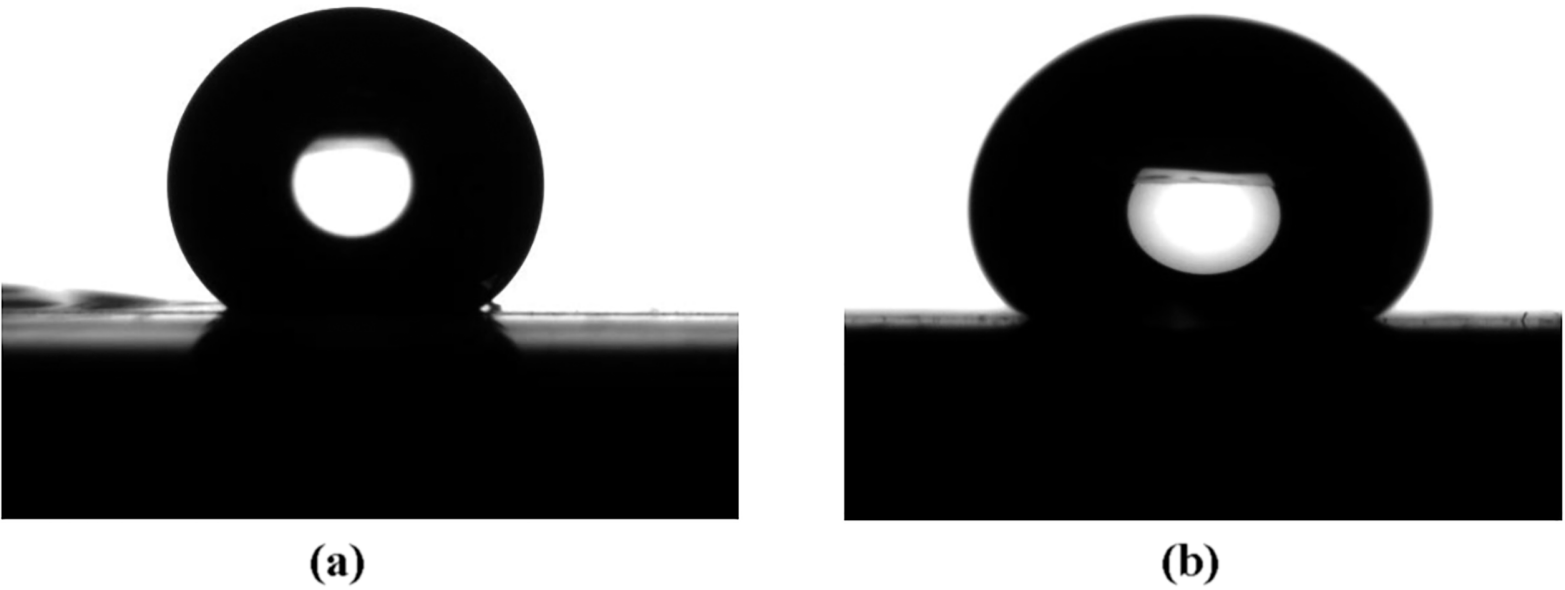
Water drop sitting on PDMS covered one scan surface of (a) glass (CA = 155.7° ± 2.6°, ROA < 2°) and (b) aluminum (CA = 156.8° ± 2.6°, ROA ∼ 3.6°).

**Figure 12:**
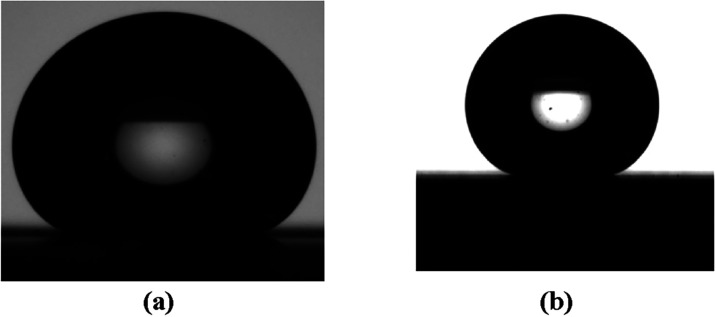
Water drop sitting on PDMS covered three scans surface of (a) glass (CA = 166.7° ± 3.4°, ROA ∼ 2°) and (b) aluminum (CA = 168.2° ± 2.6°, ROA ∼ 4.3°).

The slightly higher roll-off angle observed for the three-scan aluminum surface, despite its greater contact angle, can be attributed to the increased density and height of micro/nanostructures formed at higher laser scan numbers. Excessive surface roughness enhances air trapping and amplifies hydrophobicity but can also create localized pinning sites that temporarily anchor the droplet edges, resulting in a modest rise in the roll-off angle. Similar effects have been reported for hierarchical microtextures where overtexturing leads to partial Cassie–Wenzel transitions.^8,47^ Hence, the minor increase in roll-off angle does not signify reduced hydrophobic performance but reflects the balance between droplet adhesion and surface roughness at very high texturing densities.

### 3.3 Laser Removal of PDMS to Reverse Superhydrophobicity into Superhydrophilicity

The laser was used to ablate the PDMS coating and make the surface superhydrophilic again. The power of the laser has to be carefully chosen so that the thin PDMS layer is ablated and removed from the top of the surface features without affecting the substrate. The energy density required for this operation was found to be 1 J cm^–2^.

To evaluate the reversibility and repeatability of the process, the PDMS deposition and laser removal steps were repeated over five complete switching cycles. The measured contact angles remained consistent within ±1% variations for both aluminum and glass substrates, confirming that neither the laser texturing nor repeated PDMS processing caused degradation in wettability or surface structure. These results demonstrate that the laser-based PDMS removal method enables reliable and reproducible reversibility between superhydrophobic and superhydrophilic states.

### 3.4 Elemental Analysis of the Surface

Figures 13 and 14 present the EDS analysis results for the surface following laser microtexturing and subsequent deposition of the PDMS coating on the laser microtextured surface, respectively. The introduction of the PDMS coating leads to an elevation in the silicon and carbon percentages, attributable to the composition of PDMS as a polymer comprised of carbon and oxygen atom chains.

**Figure 13:**
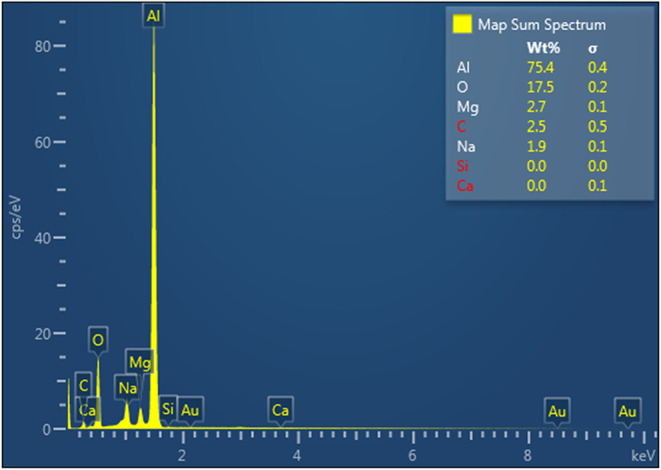
EDS analysis showing an elemental composition of the laser microtextured aluminum surface.

**Figure 14:**
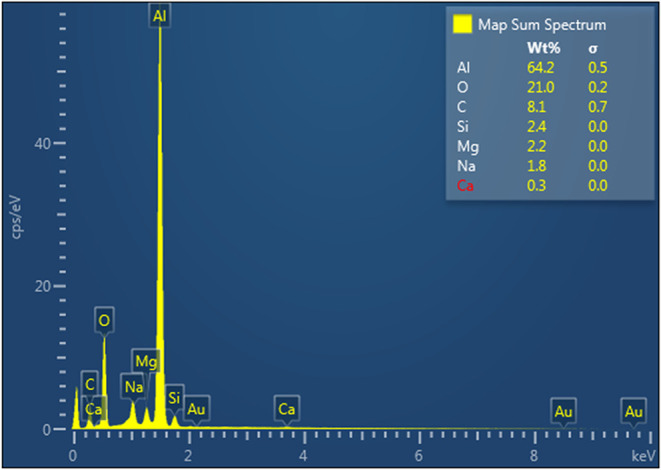
EDS analysis showing the elemental composition of the PDMS-coated laser microtextured surface.

### 3.5 Optical Characterization of PDMS-Covered Micro/Nanotextured Glass

The optical transmission of the PDMS-covered laser microtextured glass was measured to be approximately 90% throughout the wavelength range from 400 to 2500 nm. This transparency was comparable to that of plain glass, which exhibited a transmission of 92% in the same wavelength range. These findings confirm that the PDMS-covered laser microtextured glass maintains its transparency and efficiently allows the passage of light. It is important to note that a minor portion of the light is lost due to scattering induced by the surface features resulting from laser microtexturing, which is not collected by the spectrometer detector.

#### 3.5.1 Raman Spectroscopy—Substrate Quality and PDMS Coating

Raman spectra collected with 405 nm wavelength excitation show characteristic substrate signals for both aluminum and glass, confirming the initial material quality, as shown in Figure 15(a),(b). For glass, in Figure 15(a), clear glass Raman bands are observed for both the bare and laser-textured regions. However, the thin PDMS film coated textured glass shows only a weak, featureless background with no resolvable PDMS constituent peaks. For aluminum, in Figure 15(b), a Raman feature at ≈804 cm^–1^ is observed, corresponding to oxide film on the surface^48,49^ on both bare and textured samples, which persists after PDMS deposition. However, again, a broad background with no distinct PDMS peaks are detected from the thin PDMS film on textured aluminum. We attribute this to the elastic scattering from the textured substrate that masks any weak PDMS bands. Additionally, the transmission characteristic of the PDMS shows >90% transmission over the visible wavelengths.^50,51^ This leads to low absorption of incident light, which corresponds to the lack of distinct Raman peaks from the thin PDMS film. A thicker PDMS film (>500 μm) on an aluminum substrate showed the constituent Raman peaks of PDMS, as shown in Figure 15(c), which agrees with the literature.^52^ To confirm the presence of the thin PDMS film on textured samples, an FTIR measurement (in reflectance mode) was performed, which probes the film with a wavelength of >2.5 μm, where the PDMS film shows significant absorption. The FTIR reflectance measurements are shown in Figure 15(d), where the constituent reflectance peaks agree with the literature.^53^ Overall, the data indicate that (i) the substrates of aluminum and glass are spectroscopically consistent with expected material signatures, (ii) the PDMS deposited by thermal evaporation forms an ultrathin (few nanometers) film in these experiments, which can be detected by FTIR measurements.

**Figure 15:**
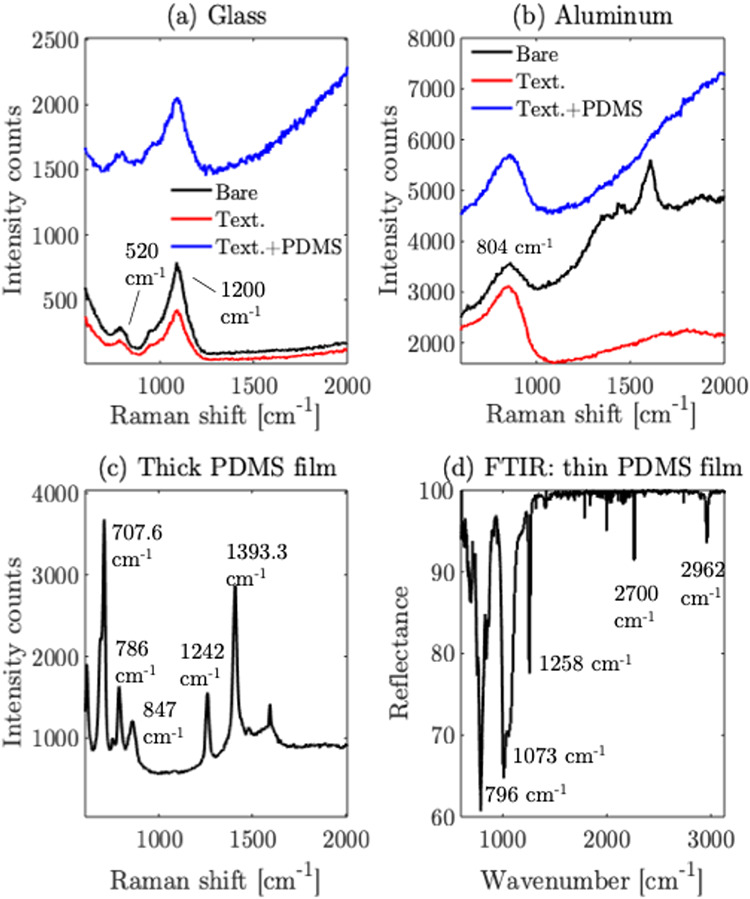
Raman measurements of (a) glass, (b) aluminum, (c) Raman spectrum of thick PDMS film, (d) FTIR spectrum of thin PDMS film on textured substrate.

These results, combined with consistent contact angle measurements across multiple regions of both aluminum and glass samples, confirm that the PDMS layer is uniformly distributed despite its nanometer-scale thickness. Following laser ablation, the complete recovery of the superhydrophilic state (contact angle <2°) and the disappearance of PDMS-specific FTIR peaks verify that the polymer film was fully removed. Thus, the spectroscopic and wettability data together confirm both the presence and successful removal of the ultrathin PDMS layer.

### 3.6 Selective Areas of Superhydrophilicity and Superhydrophobicity

A superhydrophilic–superhydrophobic patterned surface is a cohesive system that integrates both the characteristics of extreme water-repellency and water-attractiveness on a single substrate. These surfaces possess high-precision and high-resolution gradient wettability to finely control and manipulate solid–liquid interactions. These surfaces find various applications, such as droplet microfluidics, which allows precise handling of minute reagent quantities by isolating fluids in distinct droplets within cell arrays. Another application utilizes the wettability contrast between superhydrophilic and superhydrophobic patterns to control the spreading of droplets, enabling effective concentration of solutes without loss on the hydrophilic spots during surface-enhanced Raman spectroscopy of highly diluted samples. Additionally, the wettability contrast between superhydrophilic and superhydrophobic regions can be utilized to confine fluids to specific areas, offering an energy-efficient means to manipulate fluids on open surfaces, making them suitable for microfluidic devices and water harvesting.^54,55^

Figure 16 illustrates superhydrophilic surfaces represented as “squares” achieved by selectively removing PDMS from a superhydrophobic PDMS-coated aluminum surface. The dimensions of the squares shown are 375 and 200 μm; the laser ablation process allows for even smaller sizes and different shapes, offering high controllability tailored to specific applications. This level of precision sets it apart from other methods, like printing and chemical modification for creating patterned superhydrophobic-superhydrophilic surfaces.

**Figure 16:**
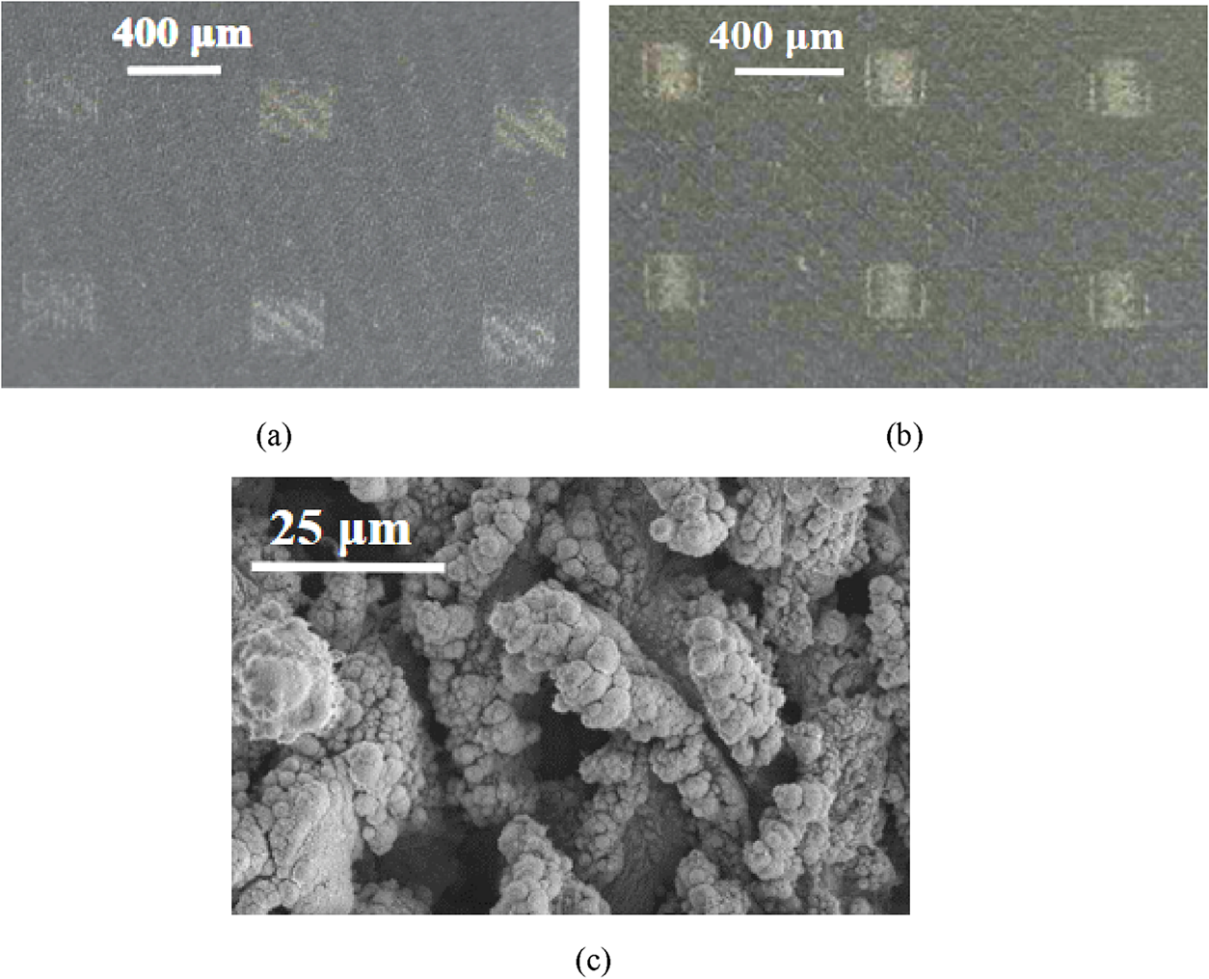
Optical microscope image of superhydrophilic squares of sizes (a) 375 μm and (b) 200 μm surrounded by superhydrophobic surface. (c) Magnified SEM image of the superhydrophilic square.

In Figure 16(c), a magnified view of one such superhydrophilic “square” reveals a micro and nanopillar arrangement that induces superhydrophilicity. Meanwhile, Figure 17 demonstrates how water drops of varying sizes are pinned on four selectively chosen superhydrophobic “squares.” This property enables accommodating and containing fluids of different volumes, which is crucial for analyzing biological fluids as described in work on nanoparticle arrays.^54^

**Figure 17:**
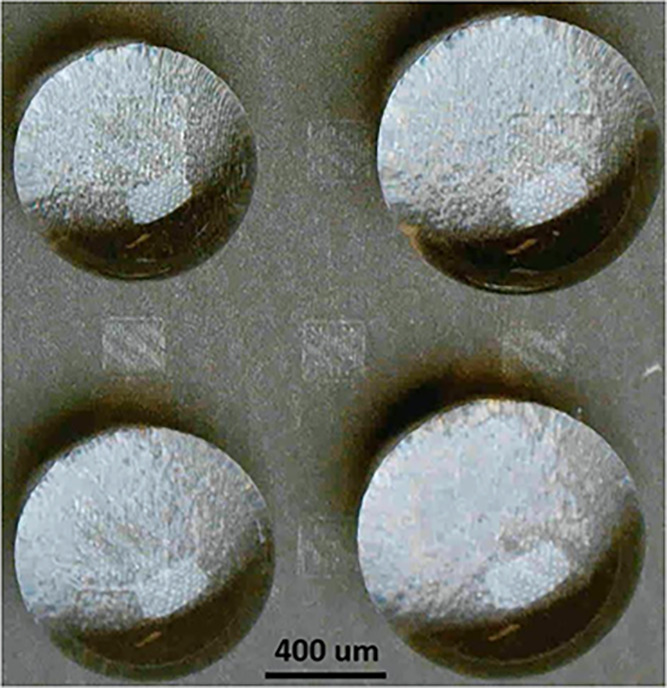
Water drops of different sizes sitting on superhydrophilic squares of size 200 μm. The volume of water drop is 10 μL (top left), 15 μL (bottom left), 25 μL (bottom right) and 35 μL (top right).

## 4 Discussion

The formation mechanism of pulsed laser-induced pillar-like microtexture involves the initial development of ripple-like structures. These ripples are believed to arise from the interference between the incident and scattered laser light at the surface, as well as various factors, including heat-mass transfer, hydrodynamic effects, and plasmonic effects. As the microstructure evolves, these ripples gradually break down, leading to the formation of micropillars. The breakdown of the ripples is primarily attributed to the expansion of molten material induced by the recoil pressure resulting from the interaction between the laser and the material.^56,57^ The features of the surface, formed through melting and ablation, are influenced by various laser parameters such as fluence, pulse width, speed, overlap of beams, and frequency. The depth and density of these features can be controlled by adjusting the fluence of the laser. Furthermore, increasing the number of laser scans leads to the ablation of more surface materials, which generates a greater depth and an increased quantity of material particles. These particles settle down on top of the microtextured features and impart nanoscale roughness.^58–60^ The formation of microscale bumps and nanoscale grains is also facilitated by the crystallization and growth of the ablated materials.^61^

The uniformity and density of surface features can be improved by scanning the surface using different orientations, such as normal (0°), orthogonal (90°), and 45° angles. Consequently, peaks and valleys are formed on the surface. In comparison to glass, the average height of surface features on aluminum is greater. As a result, the denser feature distribution on aluminum is a consequence of the ablation and resettling of a larger quantity of material particles.

The presence of densely packed and uniformly distributed surface features, along with the addition of nanoscale roughness, plays a crucial role in controlling the wettability behavior of the surface. The hierarchical surface features generated through laser microtexturing enhance surface wettability and result in a significant reduction in the contact angle (CA) value within the Wenzel region.^22^ Conversely, when the surface is coated with a low-surface energy material like PDMS, the micro/nano hierarchical surface structures contribute to achieving an exceptionally high level of water repellency.^47,62–65^ The contact angle observed on the surface with three scans is greater compared to that of the surface with only one scan. This can be attributed to the increased micro/nanoscale roughness resulting from a higher degree of ablation and resettlement of particles on the surface.

### 4.1 Mechanistic Origin of Enhanced Superhydrophilicity

The extreme wetting observed on the laser-textured substrates is likely attributable to two complementary effects produced by the picosecond 355 nm processing. First, the laser creates hierarchical surface topography. Microscale grooves and ridges decorated with nanoscale roughness substantially increases true surface area and provides capillary pathways that promote spreading of water (a Wenzel-type wetting enhancement on rough, high-energy surfaces). Second, the laser processing induces near-surface chemical modification (for example, oxidation and increased density of surface hydroxylable sites on metallic and silica-based substrates), which increases the intrinsic surface energy and favors hydrophilicity. The picosecond pulse duration and UV wavelength used here favor formation of submicron and nanotextures with limited bulk melting, producing the combination of geometrical and chemical changes that synergistically produce effectively complete wetting.

Thermal simulations using the HH2T model corroborate the experimental choice of laser fluence. The predicted surface lattice temperatures beyond 1.5 J cm^–2^ exceed the melting and ablation thresholds for aluminum and are consistent with the formation of the observed hierarchical microstructures after repeated overlapping pulses. Raman measurements with 405 nm excitation show clear substrate spectral features for glass and aluminum, while failing to reveal PDMS molecular bands for the thin evaporated coatings. However, with FTIR measurements, the thin PDMS coatings were detected. The absence of PDMS peaks is consistent with an ultrathin conformal PDMS layer and explains why PDMS can be removed with low-fluence laser ablation without significantly altering the underlying substrate morphology.

The ablation of PDMS to restore its superhydrophilicity is a crucial consideration. Ablation involves two primary mechanisms: vaporization and the expulsion of molten material from the laser focal area. The ablation threshold represents the minimum fluence value at which ablation initiates. For polymers like PDMS, this threshold typically falls within the range of 0.1 to 1 J cm^–2^. In our case, the fluence used for PDMS removal is 1 J cm^–2^. The ablation process used to remove the PDMS does not affect the metal since the ablation threshold for metals is significantly higher than that of polymers. However, using excessively high power levels could potentially lead to melting and ablation of the metal or glass surface. To avoid this, we employ a lower fluence that specifically ablates the thin PDMS layer without ablating the underlying metal.^66,67^

### 4.2 Mechanism of PDMS Removal

We interpret PDMS removal under the picosecond 355 nm exposures as predominantly a substrate/thermal-mediated process rather than bulk evaporation of PDMS. The ultrathin deposited layer is rich in volatile low-molecular-weight siloxane species, which can desorb or thermally decompose when the near-surface temperature rises rapidly. In our experiments the onset of observable removal near ≈1 J cm^–2^ and the transient near-surface heating predicted by HH2T simulations are consistent with rapid local heating sufficient to volatilize or disrupt the condensed siloxane film without substrate damage.

### 4.3 Mechanism of Wettability Transition

Laser microtexturing produces hierarchical roughness—microscale pillars topped with superimposed nanoscale features—that greatly amplifies the role of surface chemistry in determining apparent wetting, as described by the Wenzel and Cassie–Baxter models.^68^ Immediately after laser-induced textured substrates typically show superhydrophilicity for hydrophilic surfaces and superhydrophobicity for hydrophobic surfaces. Conformal deposition of a thin, low–surface-energy PDMS overlayer lowers the chemical energy of the surface so liquid no longer penetrates the texture; instead trapped air pockets remain beneath the droplet and the system switches into a composite (Cassie–Baxter) state that yields superhydrophobic contact angles with low droplet adhesion. Subsequent mild laser irradiation selectively removes the PDMS without appreciably altering the underlying microtexture, restoring the high-energy surface chemistry and returning the substrate to the hydrophilic/Wenzel regime. Thus, the reversible wettability switching observed here arises from the interplay of an unchanged laser-textured morphology (providing the physical basis and abundant capillary sites) and a reversible chemical modifier (PDMS), a mechanism consistent with prior studies of laser-textured metals and polymer coatings.^20,69,70^

The ability to spatially pattern superhydrophilic regions embedded in a superhydrophobic background (we demonstrated squares down to ∼200–375 μm) opens several microfluidic and lab-on-chip opportunities. Patterned wettability can be used to passively trap, route, merge, or split droplets without external pumps; the contrast between hydrophilic anchors and a repellent background enables droplet self-positioning, reagent metering, and droplet-based reaction sites. Similar architectures have been exploited for self-driven microfluidic devices and on-chip analyte preconcentration. This approach (laser texturing and removable PDMS) could be used to fabricate disposable, optically transparent reaction islands that are restored by PDMS removal and redeposition for repeated surface renewal. These practical avenues increase the attractiveness of the method for droplet handling, point-of-care assays, and condensation/antifogging control.^71,72^

It should be noted that different materials have different surface energies and different absorption spectra, affecting the interaction and absorption of the laser light by the material. Hence, the water flow rate will vary depending on the surface free energy of the material and the surface features. This can be used to explain the variation in the water flow rate between glass and aluminum.

We note the trade-offs of PDMS, good optical/processing compatibility but lower long-term chemical and thermal resistance compared to perfluorinated alternatives, and therefore indicate chemical-resistance and cycle-life testing as important follow-up studies for deployment in harsh environments. Finally, this laser-addressable PDMS removal approach enables fine patterned wettability (200–500 μm demonstrated), which is attractive for droplet routing, on-chip reaction islands, and reversible properties of optical windows in microfluidic and sensing applications^73,74^ while distinguishing our contribution by combining high-resolution laser patterning, PDMS overcoating, and laser recycling of PDMS on both glass and engineering alloys.

### 4.4 Practical Limits and Durability Considerations

While PDMS provides low surface energy that helps generate high apparent contact angles on laser-textured substrates, it is important to acknowledge known durability and aging limitations of PDMS-based coatings. PDMS surfaces can exhibit hydrophobic-recovery and slow changes in surface chemistry caused by migration of low-molecular-weight silicones and by surface reorientation, which can alter wettability over time.^75^ Thermal stability of PDMS is generally good compared with many organic polymers (thermal degradation reported typically above ∼250–300 °C under inert conditions), but oxidative environments and prolonged heating can modify surface properties.^76^ PDMS coatings can also be susceptible to mechanical wear. Durability is frequently improved in the literature by adding inorganic fillers (e.g., silica), or by forming PDMS-silica composites. In our present study we do not report long-term aging or abrasion data; instead, we note these as realistic limitations and as opportunities for future work. Importantly, the laser-based selective removal/regeneration approach presented here could in principle provide a localized maintenance pathway (repair or recoating of affected areas) for applications where in-service wear or local contamination limits lifetime.^77,78^ However, systematic cycling, thermal-aging, and abrasion experiments will be required to quantify lifetime and maintenance schedules for specific use-cases.

The wettability outcome of the proposed process is governed by the combined effects of surface chemistry and hierarchical roughness. On an intrinsically hydrophilic substrate, laser micro/nano texturing without chemical modification would generally enhance hydrophilicity due to increased real surface area and capillary wetting (Wenzel regime). When such a textured hydrophilic surface is further coated with a hydrophilic molecular layer, the surface is expected to remain strongly hydrophilic or even superhydrophilic. In contrast, when the same hierarchical texture is combined with a low-surface-energy coating (as in our PDMS vapor deposition), a transition toward hydrophobic or superhydrophobic behavior occurs due to air trapping and Cassie–Baxter wetting. Therefore, the technique itself is not restricted to hydrophobic outcomes; rather, it provides a tunable platform in which wettability can be engineered across hydrophilic to superhydrophobic regimes by appropriate selection of surface chemistry.

## 5 Conclusions

We demonstrated that picosecond 355 nm laser microtexturing of Al 7075 and glass produces hierarchical surface topographies that enable extreme wetting states. Laser-textured glass reached superhydrophilicity (static water contact angle <1°) and, after coating with an ultrathin (few nanometers) PDMS layer, the same textures show very high apparent hydrophobicity (static contact angles ≈ 166–168°). Controlled, localized laser exposure (energy density ≈ 1 J cm^–2^) selectively removes the PDMS overlayer and restores the underlying superhydrophilic state. Transient HH2T thermal simulations corroborate the processing window by linking absorbed fluence to near-surface heating consistent with selective PDMS removal without bulk substrate damage.

These results highlight a practical pathway for spatially resolved switching between superhydrophilic and superhydrophobic states. We emphasize that recovery of the superhydrophobic state requires redeposition of the PDMS overlayer. So, the demonstrated switching represents a laser-enabled, reconfigurable wettability platform rather than a fully autonomous or self-healing system. We note important practical limitations, in particular, known PDMS aging, thermal and wear issues, which we briefly discuss in the manuscript and which will require systematic durability testing for specific applications. Future work should therefore quantify cycle life, abrasion resistance, and thermal aging under representative service conditions, and explore composite or filler-reinforced PDMS formulations that can extend operational lifetime while preserving switchability. Overall, the combination of picosecond laser texturing, thin-film PDMS coating, and laser-enabled regeneration offers an attractive, localized maintenance strategy for surfaces where controlled wettability and in-service repair are important.
